# Supplementary material for: Chromosomal inversion polymorphisms shape the genomic landscape of deer mice
Source: Nat Ecol Evol. 2022 Oct 17;6(12):1965–79. doi: 10.1038/s41559-022-01890-0 (PMC9715431; doi:10.1038/s41559-022-01890-0)
Supplement: Supplementary file 1 — Supplementary Table 1. [file 41559_2022_1890_MOESM1_ESM.pdf]

nature ecology & evolution

Article

https://doi.org/10.1038/s41559-022-01890-0

Chromosomal inversion polymorphisms shape the genomic landscape of deer mice

In the format provided by the authors and unedited

Supplementary Table 1. List of samples for each population included in this study. Samples from populations *a – m* included in this study, listed by population from Fig. 4a, with subspecies labels for focal populations, and ecotype labels for forest and prairie populations with the following descriptors: *Sample.ID*: unique ID for each sample; *Museum.ID*: museum accession number for that sample; *Latitude/Longitude*: geographic coordinates from which sample was collected; *Source*: institution, researcher, or NCBI project from which the tissue or sequencing data was obtained (MCZ = Museum of Comparative Zoology, Harvard University; MSB = Museum of Southwestern Biology, University of New Mexico; USFS = US Forest Service; UM = University of Michigan; internal = internal from Hoekstra lab); *Sequencing*: type of sequencing data obtained for that sample (WGS = whole-genome re-sequencing; exome = exome-sequencing); *SRA.run*: NCBI SRA run identifier.

Sample.ID	Museum.ID	Latitude	Longitude	Source	Sequencing	SRA.run
**Population a**						
FSB11	MCZ69737	49.28	-122.57	MCZ	WGS	SRR20309002
FSB25	MCZ69784	49.27	-122.57	MCZ	WGS	SRR20309000
FSB37		49.28	-122.57	internal	WGS	SRR20309014
FSB42		49.28	-122.57	internal	WGS	SRR20309012
FSB43	MCZ69794	49.28	-122.57	MCZ	WGS	SRR20309010
FSB48	MCZ69756	49.27	-122.57	MCZ	WGS	SRR20309008
FSB6	MCZ69733	49.27	-122.57	MCZ	WGS	SRR20309016
FSB7	MCZ69734	49.27	-122.57	MCZ	WGS	SRR20309005
FSB51		49.27	-122.58	internal	WGS	SRR20730174
FSB52	MCZ69797	49.27	-122.58	MCZ	WGS	SRR20730172
FSB53		49.27	-122.58	internal	WGS	SRR20730171
**Population b**						
476_3_1		48.66	-110.46	Sam Cushman (USFS)	WGS	SRR20730164
477_1_4		48.66	-110.46	Sam Cushman (USFS)	WGS	SRR20730163
479_2_1		48.66	-110.46	Sam Cushman (USFS)	WGS	SRR20730161
**Population c (*P. m. rubidus*; forest ecotype)**						
ERH21	MCZ69553	44.24	-123.95	PRJNA688305	WGS	SRR13318612
ERH27	MCZ69544	44.27	-124.04	PRJNA688305	WGS	SRR13318609
ERH36	MCZ69566	44.22	-124.01	PRJNA688305	WGS	SRR13318608
ERH52	MCZ69555	44.24	-123.95	PRJNA688305	WGS	SRR13318601
ERH19	MCZ70244	44.27	-124.03	PRJNA688305	WGS	SRR13318613
ERH48	MCZ69563	44.22	-124.01	PRJNA688305	WGS	SRR13318602
ERH26	MCZ70248	44.27	-124.04	PRJNA688305	WGS	SRR13318610
ERH14	MCZ70242	44.27	-124.03	PRJNA688305	WGS	SRR13318614
ERH25	MCZ70247	44.22	-123.92	PRJNA688305	WGS	SRR13318611
ERH38		44.22	-124.01	PRJNA688305	WGS	SRR13318607
ERH42	MCZ70254	44.27	-124.03	PRJNA688305	WGS	SRR13318606
ERH12	MCZ70240	44.27	-124.04	PRJNA688305	WGS	SRR13318616
ERH43		44.22	-124.01	PRJNA688305	WGS	SRR13318605
ERH46	MCZ69562	44.22	-124.01	PRJNA688305	WGS	SRR13318603
ERH53		44.24	-123.95	PRJNA688305	WGS	SRR13318600
**Population d**						
SRR8782118	MVZ207860	37.55	-120.36	PRJNA528923	exome	SRR8782118
SRR8782119	MVZ207863	37.53	-120.35	PRJNA528923	exome	SRR8782119
SRR8782120	MVZ207864	37.54	-120.49	PRJNA528923	exome	SRR8782120
SRR8782121	MVZ207843	37.62	-120.52	PRJNA528923	exome	SRR8782121
SRR8782123	MVZ207839	37.67	-120.48	PRJNA528923	exome	SRR8782123
SRR8782191	MVZ207878	37.70	-120.24	PRJNA528923	exome	SRR8782191
SRR8782192	MVZ207847	37.51	-120.40	PRJNA528923	exome	SRR8782192
SRR8782195	MVZ207842	37.67	-120.47	PRJNA528923	exome	SRR8782195
SRR8782196	MVZ207851	37.55	-120.35	PRJNA528923	exome	SRR8782196
SRR8782197	MVZ207866	37.63	-120.21	PRJNA528923	exome	SRR8782197
SRR8782200	MVZ207870	37.66	-120.22	PRJNA528923	exome	SRR8782200
SRR8782201	MVZ207856	37.55	-120.36	PRJNA528923	exome	SRR8782201
SRR8782202	MVZ207828	37.50	-120.07	PRJNA528923	exome	SRR8782202
SRR8782205	MVZ207830	37.64	-120.15	PRJNA528923	exome	SRR8782205
SRR8782207	MVZ207832	37.67	-120.46	PRJNA528923	exome	SRR8782207
**Population e (*P. m. gambelii;* prairie ecoytpe)**						
ERH108	MCZ69536	44.85	-117.64	PRJNA688305	WGS	SRR13318593

ERH87	MCZ70287	44.81	-117.66	PRJNA688305	WGS	SRR13318653
ERH99	MCZ70296	44.81	-117.60	PRJNA688305	WGS	SRR13318667
ERH95	MCZ70292	44.81	-117.66	PRJNA688305	WGS	SRR13318700
ERH97	MCZ70294	44.81	-117.60	PRJNA688305	WGS	SRR13318678
ERH100	MCZ70297	44.81	-117.60	PRJNA688305	WGS	SRR13318615
ERH110	MCZ70302	44.81	-117.66	PRJNA688305	WGS	SRR13318725
ERH83	MCZ70286	44.85	-117.64	PRJNA688305	WGS	SRR13318714
ERH88	MCZ70288	44.82	-117.63	PRJNA688305	WGS	SRR13318642
ERH89	MCZ70289	44.82	-117.63	PRJNA688305	WGS	SRR13318631
ERH90	MCZ70290	44.85	-117.64	PRJNA688305	WGS	SRR13318758
ERH96	MCZ70293	44.81	-117.60	PRJNA688305	WGS	SRR13318689
ERH107	MCZ70300	44.82	-117.63	PRJNA688305	WGS	SRR13318604
ERH93	MCZ70291	44.81	-117.66	PRJNA688305	WGS	SRR13318747
ERH94	MCZ69534	44.85	-117.64	PRJNA688305	WGS	SRR13318736
**Population f**						
MSB280706	MSB280706	33.40	-108.59	MSB	WGS	SRR20309015
MSB280708	MSB280708	33.40	-108.59	MSB	WGS	SRR20309004
MSB280720	MSB280720	33.40	-108.58	MSB	WGS	SRR20309001
MSB280729	MSB280729	33.40	-108.58	MSB	WGS	SRR20308999
MSB280948	MSB280948	33.40	-108.58	MSB	WGS	SRR20309013
MSB280961	MSB280961	33.40	-108.58	MSB	WGS	SRR20309011
MSB280963	MSB280963	33.40	-108.58	MSB	WGS	SRR20309009
MSB280964	MSB280964	33.40	-108.58	MSB	WGS	SRR20309007
MSB280968	MSB280968	33.40	-108.59	MSB	WGS	SRR20309006
MSB280977	MSB280977	33.40	-108.58	MSB	WGS	SRR20309003
MSB280721	MSB280721	33.40	-108.58	MSB	WGS	SRR20730107
MSB280893	MSB280893	33.40	-108.58	MSB	WGS	SRR20730104
MSB280973	MSB280973	33.40	-108.58	MSB	WGS	SRR20730103
**Population g**						
SRR8782108		39.59	-105.64	PRJNA528923	exome	SRR8782108
SRR8782109		39.59	-105.64	PRJNA528923	exome	SRR8782109
SRR8782110		39.59	-105.64	PRJNA528923	exome	SRR8782110
SRR8782111		39.59	-105.64	PRJNA528923	exome	SRR8782111
SRR8782112		39.59	-105.64	PRJNA528923	exome	SRR8782112
SRR8782113		39.59	-105.64	PRJNA528923	exome	SRR8782113
SRR8782114		39.59	-105.64	PRJNA528923	exome	SRR8782114
SRR8782115		39.59	-105.64	PRJNA528923	exome	SRR8782115
SRR8782116		39.59	-105.64	PRJNA528923	exome	SRR8782116
SRR8782157		39.59	-105.64	PRJNA528923	exome	SRR8782157
SRR8782158		39.59	-105.64	PRJNA528923	exome	SRR8782158
SRR8782159		39.59	-105.64	PRJNA528923	exome	SRR8782159
SRR8782160		39.59	-105.64	PRJNA528923	exome	SRR8782160
SRR8782161		39.59	-105.64	PRJNA528923	exome	SRR8782161
SRR8782162		39.59	-105.64	PRJNA528923	exome	SRR8782162
SRR8782165		39.59	-105.64	PRJNA528923	exome	SRR8782165
SRR8782166		39.59	-105.64	PRJNA528923	exome	SRR8782166
SRR8782167		39.59	-105.64	PRJNA528923	exome	SRR8782167
SRR8782169		39.59	-105.64	PRJNA528923	exome	SRR8782169
SRR8782170		39.59	-105.64	PRJNA528923	exome	SRR8782170
SRR8782171		39.59	-105.64	PRJNA528923	exome	SRR8782171
SRR8782172		39.59	-105.64	PRJNA528923	exome	SRR8782172
SRR8782173		39.59	-105.64	PRJNA528923	exome	SRR8782173
SRR8782174		39.59	-105.64	PRJNA528923	exome	SRR8782174
SRR8782175		39.59	-105.64	PRJNA528923	exome	SRR8782175
SRR8782176		39.59	-105.64	PRJNA528923	exome	SRR8782176
SRR8782177		39.59	-105.64	PRJNA528923	exome	SRR8782177
SRR8782178		39.59	-105.64	PRJNA528923	exome	SRR8782178
SRR8782179		39.59	-105.64	PRJNA528923	exome	SRR8782179
SRR8782180		39.59	-105.64	PRJNA528923	exome	SRR8782180
SRR8782181		39.59	-105.64	PRJNA528923	exome	SRR8782181
SRR8782182		39.59	-105.64	PRJNA528923	exome	SRR8782182
SRR8782183		39.59	-105.64	PRJNA528923	exome	SRR8782183
SRR8782184		39.59	-105.64	PRJNA528923	exome	SRR8782184
SRR8782185		39.59	-105.64	PRJNA528923	exome	SRR8782185
SRR8782186		39.59	-105.64	PRJNA528923	exome	SRR8782186
SRR8782187		39.59	-105.64	PRJNA528923	exome	SRR8782187

SRR8782188		39.59	-105.64	PRJNA528923	exome	SRR8782188
SRR8782189		39.59	-105.64	PRJNA528923	exome	SRR8782189
SRR8782190		39.59	-105.64	PRJNA528923	exome	SRR8782190
SRR8782193		39.59	-105.64	PRJNA528923	exome	SRR8782193
SRR8782194		39.59	-105.64	PRJNA528923	exome	SRR8782194
SRR8782199		39.59	-105.64	PRJNA528923	exome	SRR8782199
SRR8782203		39.59	-105.64	PRJNA528923	exome	SRR8782203
SRR8782204		39.59	-105.64	PRJNA528923	exome	SRR8782204
SRR8782206		39.59	-105.64	PRJNA528923	exome	SRR8782206
**Population h**						
SRR8782117		40.81	-96.68	PRJNA528923	exome	SRR8782117
SRR8782122		40.81	-96.68	PRJNA528923	exome	SRR8782122
SRR8782124		40.81	-96.68	PRJNA528923	exome	SRR8782124
SRR8782125		40.81	-96.68	PRJNA528923	exome	SRR8782125
SRR8782126		40.81	-96.68	PRJNA528923	exome	SRR8782126
SRR8782127		40.81	-96.68	PRJNA528923	exome	SRR8782127
SRR8782128		40.81	-96.68	PRJNA528923	exome	SRR8782128
SRR8782129		40.81	-96.68	PRJNA528923	exome	SRR8782129
SRR8782130		40.81	-96.68	PRJNA528923	exome	SRR8782130
SRR8782131		40.81	-96.68	PRJNA528923	exome	SRR8782131
SRR8782132		40.81	-96.68	PRJNA528923	exome	SRR8782132
SRR8782133		40.81	-96.68	PRJNA528923	exome	SRR8782133
SRR8782134		40.81	-96.68	PRJNA528923	exome	SRR8782134
SRR8782135		40.81	-96.68	PRJNA528923	exome	SRR8782135
SRR8782136		40.81	-96.68	PRJNA528923	exome	SRR8782136
SRR8782137		40.81	-96.68	PRJNA528923	exome	SRR8782137
SRR8782139		40.81	-96.68	PRJNA528923	exome	SRR8782139
SRR8782141		40.81	-96.68	PRJNA528923	exome	SRR8782141
SRR8782142		40.81	-96.68	PRJNA528923	exome	SRR8782142
SRR8782143		40.81	-96.68	PRJNA528923	exome	SRR8782143
SRR8782144		40.81	-96.68	PRJNA528923	exome	SRR8782144
SRR8782145		40.81	-96.68	PRJNA528923	exome	SRR8782145
SRR8782146		40.81	-96.68	PRJNA528923	exome	SRR8782146
SRR8782147		40.81	-96.68	PRJNA528923	exome	SRR8782147
SRR8782148		40.81	-96.68	PRJNA528923	exome	SRR8782148
SRR8782149		40.81	-96.68	PRJNA528923	exome	SRR8782149
SRR8782150		40.81	-96.68	PRJNA528923	exome	SRR8782150
SRR8782151		40.81	-96.68	PRJNA528923	exome	SRR8782151
SRR8782152		40.81	-96.68	PRJNA528923	exome	SRR8782152
SRR8782153		40.81	-96.68	PRJNA528923	exome	SRR8782153
SRR8782154		40.81	-96.68	PRJNA528923	exome	SRR8782154
SRR8782155		40.81	-96.68	PRJNA528923	exome	SRR8782155
SRR8782156		40.81	-96.68	PRJNA528923	exome	SRR8782156
SRR8782163		40.81	-96.68	PRJNA528923	exome	SRR8782163
SRR8782164		40.81	-96.68	PRJNA528923	exome	SRR8782164
**Population i**						
JJK_4190	MCZ70690	38.30	-84.51	MCZ	WGS	SRR20730162
JJK_4191	MCZ70691	38.30	-84.51	MCZ	WGS	SRR20730151
JJK_4192	MCZ70692	38.30	-84.51	MCZ	WGS	SRR20730140
**Population j (*P. m. bairdii*)**						
MZ10760		42.20	-83.89	Cody Thompson (UM)	WGS	SRR20730094
MZ10762		42.20	-83.89	Cody Thompson (UM)	WGS	SRR20730092
MZ10764		42.20	-83.89	Cody Thompson (UM)	WGS	SRR20730091
MZ10770		42.20	-83.89	Cody Thompson (UM)	WGS	SRR20730090
MZ10772		42.20	-83.89	Cody Thompson (UM)	WGS	SRR20730089
MZ10774		42.20	-83.89	Cody Thompson (UM)	WGS	SRR20730088
MZ10804		42.20	-83.89	Cody Thompson (UM)	WGS	SRR20730087
MZ10808		42.20	-83.89	Cody Thompson (UM)	WGS	SRR20730214
MZ10812		42.20	-83.89	Cody Thompson (UM)	WGS	SRR20730213
MZ10813		42.20	-83.89	Cody Thompson (UM)	WGS	SRR20730211
MZ10814		42.20	-83.89	Cody Thompson (UM)	WGS	SRR20730210
MZ10815		42.20	-83.89	Cody Thompson (UM)	WGS	SRR20730209
MZ10884		42.20	-83.89	Cody Thompson (UM)	WGS	SRR20730208
MZ10885		42.20	-83.89	Cody Thompson (UM)	WGS	SRR20730207
MZ11010		42.20	-83.89	Cody Thompson (UM)	WGS	SRR20730206
MZ11011		42.20	-83.89	Cody Thompson (UM)	WGS	SRR20730205

MZ11012		42.20	-83.89	Cody Thompson (UM)	WGS	SRR20730204
**Population k**						
EPK001		45.41	-84.16	internal	WGS	SRR20730048
EPK002		45.41	-84.16	internal	WGS	SRR20730047
EPK003		45.41	-84.16	internal	WGS	SRR20730046
EPK004		45.41	-84.16	internal	WGS	SRR20730045
EPK005		45.41	-84.16	internal	WGS	SRR20730044
EPK006		45.41	-84.16	internal	WGS	SRR20730043
EPK007		45.41	-84.16	internal	WGS	SRR20730042
EPK008		45.41	-84.16	internal	WGS	SRR20730041
EPK010		45.41	-84.16	internal	WGS	SRR20730039
EPK011		45.41	-84.16	internal	WGS	SRR20730038
EPK012		45.41	-84.16	internal	WGS	SRR20730037
EPK014		45.41	-84.16	internal	WGS	SRR20730036
EPK017		45.41	-84.16	internal	WGS	SRR20730035
EPK018		45.41	-84.16	internal	WGS	SRR20730034
EPK019		45.41	-84.16	internal	WGS	SRR20730033
**Population l (*P. m. nubiterrae*)**						
EPK04		40.33	-79.27	internal	WGS	SRR19238025
EPK14		40.33	-79.27	internal	WGS	SRR20730135
EPK07		40.33	-79.27	internal	WGS	SRR20730134
EPK11		40.33	-79.27	internal	WGS	SRR20730133
EPK17		40.33	-79.27	internal	WGS	SRR20730132
EPK08		40.33	-79.27	internal	WGS	SRR20730131
EPK02		40.33	-79.27	internal	WGS	SRR20730130
EPK03		40.33	-79.27	internal	WGS	SRR20730128
EPK01		40.33	-79.27	internal	WGS	SRR20730127
EPK15		40.33	-79.27	internal	WGS	SRR20730126
EPK10		40.33	-79.27	internal	WGS	SRR20730125
EPK18		40.33	-79.27	internal	WGS	SRR20730124
EPK09		40.33	-79.27	internal	WGS	SRR20730123
EPK16		40.33	-79.27	internal	WGS	SRR20730122
NUB13		40.33	-79.27	internal	WGS	SRR20730121
**Population m (*P. p. subgriseus*)**						
68144	MCZ68144	29.18	-81.80	MCZ	WGS	SRR19237879
68146	MCZ68146	29.18	-81.80	MCZ	WGS	SRR19237877
68147	MCZ68147	29.18	-81.80	MCZ	WGS	SRR19237876
68148	MCZ68148	29.18	-81.80	MCZ	WGS	SRR19237875
68149	MCZ68149	29.18	-81.80	MCZ	WGS	SRR19237874
68150	MCZ68150	29.18	-81.80	MCZ	WGS	SRR19237873
68151	MCZ68151	29.18	-81.80	MCZ	WGS	SRR19237872
68152	MCZ68152	29.18	-81.80	MCZ	WGS	SRR19237871
68153	MCZ68153	29.18	-81.80	MCZ	WGS	SRR19237870
68154	MCZ68154	29.18	-81.80	MCZ	WGS	SRR19237869
68159	MCZ68159	29.18	-81.80	MCZ	WGS	SRR19237868
68160	MCZ68160	29.18	-81.80	MCZ	WGS	SRR19237866
68161	MCZ68161	29.18	-81.80	MCZ	WGS	SRR19237961
68162	MCZ68162	29.18	-81.80	MCZ	WGS	SRR19237960
68163	MCZ68163	29.18	-81.80	MCZ	WGS	SRR19237959
P. leucopus						
MSB_212419	MSB212419	36.10	-94.00	MSB	WGS	SRR20730233
MSB_212420	MSB212420	36.10	-94.00	MSB	WGS	SRR20730232
MSB_212421	MSB212421	36.10	-94.00	MSB	WGS	SRR20730106
MSB_53354	MSB53354	46.90	-96.50	MSB	WGS	SRR20730167
MSB_53355	MSB53355	46.90	-96.50	MSB	WGS	SRR20730166
MSB_53357	MSB53357	46.90	-96.50	MSB	WGS	SRR20730165
MSB_229927	MSB229927	43.60	-73.70	MSB	WGS	SRR20730144
MSB_229967	MSB229967	43.60	-73.70	MSB	WGS	SRR20730143
MSB_42375	MSB42375	33.85	-100.79	MSB	WGS	SRR20730115
MSB_42378	MSB42378	33.85	-100.79	MSB	WGS	SRR20730114
MSB_42402	MSB42402	33.85	-100.79	MSB	WGS	SRR20730113
P. californicus						
47_IS_F_17437		NA	NA	internal	WGS	SRR20730224
IS_SRA		NA	NA	PRJNA53595	WGS	SAMN01081713
