# Supplementary material for: Chromosomal inversion polymorphisms shape the genomic landscape of deer mice
Source: Nat Ecol Evol. 2022 Oct 17;6(12):1965–79. doi: 10.1038/s41559-022-01890-0 (PMC9715431; doi:10.1038/s41559-022-01890-0)
Supplement: Supplementary file 2 — Reporting Summary [file 41559_2022_1890_MOESM2_ESM.pdf]

nature portfolio

Corresponding author(s): Olivia Harringmeyer, Hopi Hoekstra

Last updated by author(s): Jul 28, 2022

Reporting Summary

Nature Portfolio wishes to improve the reproducibility of the work that we publish. This form provides structure for consistency and transparency in reporting. For further information on Nature Portfolio policies, see our Editorial Policies and the Editorial Policy Checklist.

Statistics

For all statistical analyses, confirm that the following items are present in the figure legend, table legend, main text, or Methods section.

n/a	Confirmed	
☐	☒	The exact sample size (*n*) for each experimental group/condition, given as a discrete number and unit of measurement
☐	☒	A statement on whether measurements were taken from distinct samples or whether the same sample was measured repeatedly
☐	☒	The statistical test(s) used AND whether they are one- or two-sided<br>*Only common tests should be described solely by name; describe more complex techniques in the Methods section.*
☐	☒	A description of all covariates tested
☐	☒	A description of any assumptions or corrections, such as tests of normality and adjustment for multiple comparisons
☐	☒	A full description of the statistical parameters including central tendency (e.g. means) or other basic estimates (e.g. regression coefficient) AND variation (e.g. standard deviation) or associated estimates of uncertainty (e.g. confidence intervals)
☐	☒	For null hypothesis testing, the test statistic (e.g. F, t, r) with confidence intervals, effect sizes, degrees of freedom and P value noted<br>*Give P values as exact values whenever suitable.*
☒	☐	For Bayesian analysis, information on the choice of priors and Markov chain Monte Carlo settings
☒	☐	For hierarchical and complex designs, identification of the appropriate level for tests and full reporting of outcomes
☒	☐	Estimates of effect sizes (e.g. Cohen's *d*, Pearson's *r*), indicating how they were calculated

Our web collection on statistics for biologists contains articles on many of the points above.

Software and code

Policy information about availability of computer code

Data collection	Short-read whole-genome sequencing was performed using Illumina NovaSeq. Long-read whole-genome sequencing was performed using PacBio Sequel II.
Data analysis	BWA v0.7.17 was used for read-mapping, and GATK v3.8 was used for variant calling. Lostruct (https://github.com/petrelharp/local_pca) was used for local PCA. Population genomic analyses were performed using scikit-allel v1.3.2 (https://github.com/cggh/scikit-allel). bcftools v1.5 and vcftools v0.1.15 were used for variant filtering. Linkage disequilibrium was computed using the script emerald2windowldcounts.pl (https://github.com/owensgl/reformat, https://github.com/owensgl/haploblocks). Genome assembly was performed using flye v2.8.3 (https://github.com/fenderglass/Flye). Variant calling from long-read data was performed with ngmlr (https://github.com/philres/ngmlr) and longshot (https://github.com/pjedge/longshot). Mummer v3 was used for genome alignments. SEDEF (https://github.com/vpc-ccg/sedef) was used for detecting segmental duplications. RepeatMasker v4.0.5 was used to identify and mask common repeats. Phylogenetic trees were created with RAxML v8.2.12, with data prepared using vcf2phylip.py (https://github.com/edgardomortiz/vcf2phylip) and ascbias.py (https://github.com/btmartin721/raxml_ascbias). The genetics (https://cran.r-project.org/web/packages/genetics/index.html) package in R was used to analyze genotype frequencies. PopGenome v2.7.5 was used for mutational load analyses. SLiM v3.6 was used for forward-genetic simulations. HZAR v0.2.5 was used for clinal analyses. Additional data analyses and plotting were performed in R v3.4.2.

For manuscripts utilizing custom algorithms or software that are central to the research but not yet described in published literature, software must be made available to editors and reviewers. We strongly encourage code deposition in a community repository (e.g. GitHub). See the Nature Portfolio guidelines for submitting code & software for further information.

Data

Policy information about availability of data

All manuscripts must include a data availability statement. This statement should provide the following information, where applicable:

- Accession codes, unique identifiers, or web links for publicly available datasets
- A description of any restrictions on data availability
- For clinical datasets or third party data, please ensure that the statement adheres to our policy

Sequencing data is available from NCBI SRA (project numbers PRJNA856879, PRJNA816517, PRJNA860096, PRJNA862503).

Field-specific reporting

Please select the one below that is the best fit for your research. If you are not sure, read the appropriate sections before making your selection.

☒ Life sciences ☐ Behavioural & social sciences ☐ Ecological, evolutionary & environmental sciences

For a reference copy of the document with all sections, see nature.com/documents/nr-reporting-summary-flat.pdf

Life sciences study design

All studies must disclose on these points even when the disclosure is negative.

Sample size	Sample sizes for short-read whole genome re-sequencing (n=15-17 samples/population, 15x coverage) enable genotype calls and estimates of population genomic parameters at reasonable costs. Sample sizes for long-read sequencing (n=1 sample/population) were chosen based on feasibility.
Data exclusions	No data were excluded from this study.
Replication	All analyses pipelines are fully described in the methods, and both associated data and code are provided.
Randomization	Randomization is not relevant for this study because experimental groups were not compared in this study.
Blinding	Blinding is not relevant for this study because samples were not assigned to experimental groups.

Reporting for specific materials, systems and methods

We require information from authors about some types of materials, experimental systems and methods used in many studies. Here, indicate whether each material, system or method listed is relevant to your study. If you are not sure if a list item applies to your research, read the appropriate section before selecting a response.

Materials & experimental systems

n/a	Involved in the study	
☒	☐	Antibodies
☒	☐	Eukaryotic cell lines
☒	☐	Palaeontology and archaeology
☐	☒	Animals and other organisms
☒	☐	Human research participants
☒	☐	Clinical data
☒	☐	Dual use research of concern

Methods

n/a	Involved in the study	
☒	☐	ChIP-seq
☒	☐	Flow cytometry
☒	☐	MRI-based neuroimaging

Animals and other organisms

Policy information about studies involving animals; ARRIVE guidelines recommended for reporting animal research

Laboratory animals	Five laboratory mice (4 Peromyscus maniculatus, 1 Peromyscus polionotus, approximately 60-100 days old) were used in this study for long-read sequencing. One laboratory mouse (Peromyscus californicus) was used in this study for short-read whole-genome sequencing.
Wild animals	Wild-caught specimens and tissues used in this study were museum accessioned or obtained from previous publications.
Field-collected samples	This study did not involve samples directly collected from the field.

Ethics oversight | Harvard Unviersity's IACUC approved of the experiments of this study.

Note that full information on the approval of the study protocol must also be provided in the manuscript.
